# Targeted Enlargement of Aptamer Functionalized Gold Nanoparticles for Quantitative Protein Analysis

**DOI:** 10.3390/proteomes5010001

**Published:** 2016-12-22

**Authors:** Feng Li, Jingjing Li, Yanan Tang, Chuan Wang, Xing-Fang Li, X. Chris Le

**Affiliations:** Department of Laboratory Medicine and Pathology, University of Alberta, Edmonton, AB T6G 2G3, Canada; qingchao0124@163.com (J.L.); yanan3@ualberta.ca (Y.T.); chuan2@ualberta.ca (C.W.); xingfang@ualberta.ca (X.-F.L.)

**Keywords:** aptamer, protein quantification, Western blot, gold enlargement

## Abstract

The ability to selectively amplify the detection signals for targets over interferences is crucial when analyzing proteins in a complicated sample matrix. Here, we describe a targeted enlargement strategy that can amplify the light-scattering signal from aptamer-functionalized gold nanoparticles (Apt-AuNP) with high specificity for quantitative protein analysis. This strategy is achieved by labeling target proteins with competitively protected Apt-AuNP probes and enlarging the probes with gold enhancement. This competitive protection strategy could effectively eliminate nonspecific protein adsorptions from a sample matrix, leading to a highly specific labeling of the target protein. As a result, the subsequent amplification of the light-scattering signal by gold enhancement only occurs in the presence of the target protein. This strategy was successfully demonstrated by analyzing human α-thrombin in human serum samples in a Western blot format.

## 1. Introduction

While the discovery-based proteomic strategies profile protein expressions, interactions, and pathways from a biological sample at global levels, targeted protein or proteome analysis is hypothesis-driven and focuses on a subgroup of proteins of interest [1]. Although strategies that are enabled by mass spectrometry and bioinformatics, such as selected reaction monitoring (SRM) and multiple reaction monitoring (MRM), are revolutionizing the field of targeted protein and proteome research, techniques making use of polyacrylamide gels and immunomembranes, including sodium dodecyl sulfate polyacrylamide gel electrophoresis (SDS-PAGE), Two Dimensional (2D)-gel electrophoresis, and Western blotting are still the major workhorses in most biochemical laboratories for the quantitative protein analyses [2]. Therefore, it is worthwhile to develop novel molecular probes that are sensitive and robust for the detection of immunoblotted proteins. Here, we describe a facile signal amplification strategy for immunoblotted proteins via the use of aptamer-functionalized gold nanoparticles (Apt-AuNPs). 

AuNPs have shown great promise and exciting opportunities for biomolecular analysis due to their unique resonance light-scattering properties and versatility for bioconjugations [3]. Compared to conventionally-used fluorescent or luminescent labels, AuNPs offer high light-scattering intensities, quenching and photo-bleaching resistance, and colorimetric signal read-out that enables robust detection of target molecules [4,5]. These advantages have spurred the development of diverse AuNP-based assays for the detection of nucleic acids [6,7,8], proteins [9,10,11,12,13], pathogens [14], and cancer cells [15]. However, surfaces of AuNPs are known to cause strong nonspecific binding via electrostatic and hydrophobic interactions [16,17]. Consequently, when analyzing targets in complicated sample matrixes, e.g., human serum, any nonspecifically adsorbed AuNP labels may contribute to backgrounds, leading to possible false positive results and compromising detection sensitivity [12,18]. Thus, it is essential to reduce or eliminate nonspecific bindings when applying AuNPs for analyzing targets in complicated sample matrixes. To achieve this goal, we have previously developed a competitive protection strategy that is able to effectively eliminate nonspecific bindings between aptamer functionalized AuNPs (Apt-AuNP) and interference human serum proteins by controlling the DNA assembly on Apt-AuNPs [18]. Here, taking advantage of the competitive protection strategy, we further develop a targeted enlargement method that is able to amplify the light-scattering signal from the protected Apt-AuNP probes by catalytic deposition of metal onto AuNPs for colorimetric analysis of human serum samples in a Western blot format. 

Catalytic deposition of metal onto AuNPs, e.g., silver enhancement or gold enhancement, has been widely used as signal amplification strategies in scanometric assays and other colorimetric immunoassays [6,9,10,11,12,13,14]. It has been demonstrated that the light-scattering intensity can be amplified over 5 orders of magnitude by enlarging AuNP labels with silver or gold enhancement [19]. However, the high amplification capacity is also challenged by the strong background raised from nonspecifically bound AuNPs to interferences associated with the target. Here, we reason that the competitive protection strategy can overcome this challenge by effectively eliminating nonspecific adsorptions of Apt-AuNPs to interferences, leading to a targeted enlargement of Apt-AuNPs specifically to the minute amount of target proteins in a complicated sample matrix. 

## 2. Materials and Methods 

### 2.1. Materials 

Human α-thrombin was purchased from Haematologic Technologies Inc. (Essex Junction, VT, USA). Solution of AuNPs (10 nm in diameter), Bovine serum albumin (BSA), human serum (product number, P2918) and tris (2-carboxyethyl) phosphine hydrochloride (TCEP) were purchased from Sigma (Sigma-Aldrich Co. LLC., Oakville, ON, Canada). The aptamer for human α-thrombin (5′-SH-(CH2)6-GGT TGG TGT GGT TGG-3′, and 5′-FAM-GGT TGG TGT GGT TGG-3′), and protection DNA PolyA-Oligo10 (5′-AAA AAA AAA AAA AAA AAA AAC CAA CCA CAC-3′), were synthesized and purified by Integrated DNA Technologies Inc. (IDT, Coralville, IA, USA). LI SilverTM enhancement kit) and GoldTM enhancement kit were purchased from Nanoprobes, Inc. (Yaphank, NY, USA). Reagents for SDS-PAGE gel electrophoresis and Western blot were purchased from BioRad Laboratories (BioRad Laboratories LTD. Mississauga, ON, Canada) and Fisher Scientific (Thermo Fisher Scientific Inc. Mississauga, ON, Canada). The binding buffer contained 100 mM NaCl, 20 mM Tris-HCl, 2 mM MgCl, 5 mM KCl, 1 mM CaCl2, and 0.02% Tween 20. The washing buffer contained 1× phosphate buffer saline (PBS), and 0.1% Tween 20, pH 7.4. The protein loading buffer (10 mL) was made of 800 mg SDS, 4.0 mL glycerol, 0.4 mL 2-mercaptoethanol, 2.0 mL Tris-HCl at pH 6.8, and 8 mg bromophenol blue.

### 2.2. Preparation and Characterization of Apt-AuNPs and Protected Apt-AuNPs

A thiolated aptamer probe was received in a disulfide form. Prior to use, it was activated by 50 μL of 5 mM TCEP in 100 mM Tris-HCl for 1 h at room temperature. This solution was then added to 1 mL AuNPs solution, and the mixture was placed at 4 °C for 16 h. To this mixture was slowly added 100 μL of 2 M NaCl, and followed by 10 s sonication. This process was repeated five times with 20 min intervals to maximize the aptamer loading amounts. The final solution was stored at 4 °C for 24 h. Then, it was centrifuged at 17,000× *g* for 1 h to separate the AuNPs from the unreacted reagents. The AuNPs were washed three times with 10 mM Tris-HCl (pH 7.4), and then redispersed in 1 mL of 10 mM Tris-HCl (pH 7.4). The aptamer modified AuNPs solution was stored at 4 °C when not in use and was found to be stable for more than two weeks.

To prepare the protected Apt-AuNPs, Apt-AuNPs were treated using the following protocol. Protection DNA (PolyA-oligo10) was added to Apt-AuNP solution, and the final concentrations of both the aptamer and the protection oligo were kept to 1 μM. The aptamer and protection DNA in the mixture were allowed to hybridize for 1.5 h at room temperature. The solution was then diluted 10 times with binding buffer for Western blot applications.

**Figure 1:**
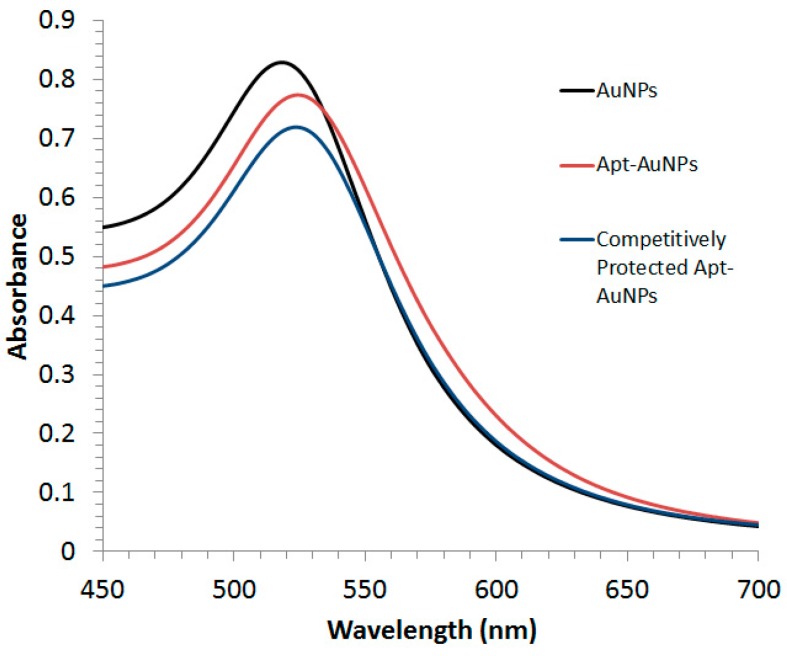
Characterization of gold nanoparticles (AuNPs), aptamer-functionalized gold nanoparticles (Apt-AuNPs), and competitively protected Apt-AuNPs using a UV/Vis Spectrophotometer. The maximum absorbance was observed to be 525 nm for bare AuNPs (black curve) and the modification of AuNPs with DNA oligonucleotides led to a 4-nm red-shift in the absorbance spectra (red and blue curves).

Both Apt-AuNPs and protected Apt-AuNPs were characterized by UV/Vis absorption spectrometry (Figure 1). A Lambda 35 UV/Vis absorption spectrophotometer (PerkinElmer LAS Canada INC., Woodbridge, ON, Canada) was used to collect extinction spectra of Apt-AuNPs and protected Apt-AuNPs from 400 nm to 700 nm. 

### 2.3. Gel Electrophoresis, Protein Transferring, and Membrane Blocking

SDS-PAGE separation of proteins was performed with 5% stacking gel and 12% resolving gel. All the gels were freshly prepared in house. Before loading, protein samples were mixed with protein loading buffer on a volume ratio of 3:1 and then heated at 95 °C for 5 min. A potential of 12 V/cm was applied for gel electrophoresis separation. The proteins on the gel were then transferred to the Polyvinylidene fluoride (PVDF) membrane with a constant voltage of 120 V for 1 h. During this procedure, the temperature was kept at 4 °C. The blotting buffer (1000 mL) consisted of 200 mL methanol, 100 mL 10× Tris-glycine buffer, and 700 mL deionized water. After transferring, the gel was stained with Coomassie Brilliant Blue R-250 (BioRad Laboratories LTD.) to estimate the efficiency of protein transferring (~60% in our experiment). The membrane was immediately blocked with 3% BSA for 1 h to prevent nonspecific binding. After blocking, the membrane was washed twice with washing buffer for 10 min each and once with binding buffer. For serum samples, the total protein amount was determined by measuring absorbance at 280 nm.

### 2.4. Protein Detection by Apt-AuNPs and Silver/Gold Enhancement of Apt-AuNPs

After gel electrophoresis, transferring and membrane blocking steps, Apt-AuNP probes were added and incubated for 1 h at room temperature. After incubation, the membrane was washed three times with washing buffer (10 min each time) and dried at room temperature. The Apt-AuNPs stained protein bands on the membrane can be directly visualized by the naked eye. To quantify the protein amount, the dried membrane was scanned by a desktop flatbed scanner (hp scanjet 4400c) and analyzed by imaging software (Adobe Photoshop CS3). For further signal enhancement, commercial silver or gold enhancement kits were used, following the recommended protocols for immune-membranes.

### 2.5. Signal Amplification Using Targeted Enlargement of Apt-AuNPs

For signal amplification using targeted enlargement of Apt-AuNPs, protected Apt-AuNP probes were used to stain the target protein on membrane. After washing three times, the membrane containing the target protein and protected Apt-AuNPs was soaked in gold enhancement solution for 5 min, washed three times with deionized water, and left to dry. The dried membrane was imaged by a desktop flatbed scanner and analyzed by imaging software.

## 3. Results and Discussion

**Scheme 1:**
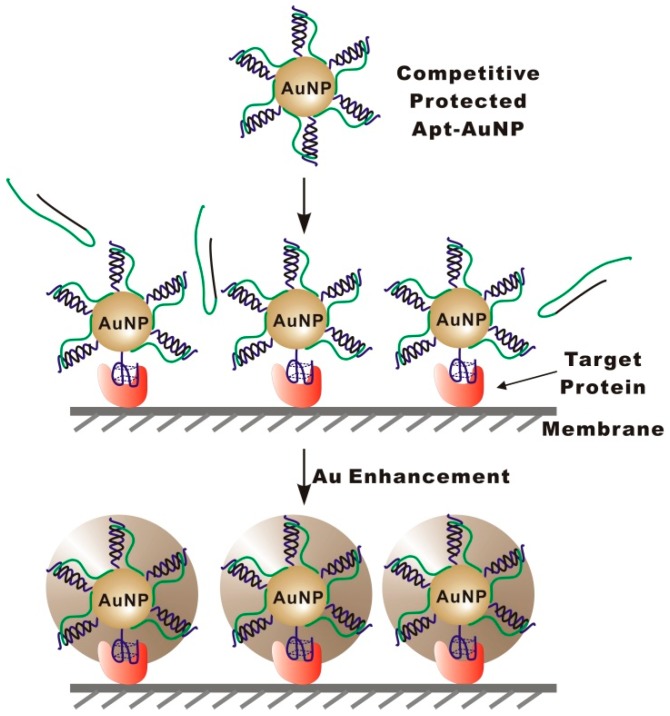
Schematic showing the targeted enlargement of aptamer (blue line) functionalized gold nanoparticles (Apt-AuNPs) for immunoblotting-based protein detection. To achieve the targeted enlargement, Apt-AuNPs are protected with protection DNA oligonucleotides (black line is the complementary region for the aptamer strand and green line is the polyA protection region) before being applied to target proteins. The protection DNA restricts the access of interfering molecules to the AuNP surface through a polyA overhang, thereby eliminating nonspecific interactions. After labeling the target protein with the protected Apt-AuNPs, a subsequent gold enhancement is used to enlarge the Apt-AuNPs that have been specifically captured by the target protein, enabling the colorimetric detection of the target protein.

The strategy of targeted enlargement of Apt-AuNPs is shown in Scheme 1. To achieve the targeted enlargement, we construct competitively protected Apt-AuNP probes by assembling protection DNA (PolyA-Oligo10) onto Apt-AuNPs through a complementary sequence to the conjugated aptamer. Once assembled on AuNPs, the protection DNA could restrict the access of interfering molecules to the AuNP surface through a polyA overhang, thereby eliminating nonspecific interactions. In detection of the target protein that is either blotted or captured on a solid substrate, e.g., immunomembrane or microplate well (in ELISA assay format), competitive binding of the target protein to the aptamer results in a substitution of the protection DNA, leading to the specific labeling of the target protein with Apt-AuNPs. Non-target molecules could not displace the protection DNA because their binding to aptamers is much weaker than the hybridization between the protection DNA and aptamer sequence. After specifically labeling the target protein with Apt-AuNPs, a subsequent gold enhancement is performed to enlarge the labeled Apt-AuNPs. As a result of the targeted enlargement of Apt-AuNPs, the light-scattering signal can then be amplified, enabling the sensitive detection of the target protein.

To demonstrate the feasibility of our strategy, we chose human α-thrombin as a target protein because of its important role in the blood clotting process. A 15-mer DNA aptamer that could bind to the fibrinogen site of thrombin was used as an affinity ligand. To evaluate the specificity of our strategy for detecting target protein specifically in a complicated sample matrix, we spiked different concentrations of thrombin into human serum samples, and then separated thrombin from interfering serum proteins by gel electrophoresis followed by the transfer of all proteins to a polyvinylidene fluoride (PVDF) membrane using electroblotting (Western blot). The PVDF membrane was chosen because of its wide application in Western blot. By separately blotting the target thrombin and interfering serum proteins onto PVDF membranes, we can easily evaluate the performance of our strategy by comparing the intensities of different protein bands on the same membrane.

**Figure 2:**
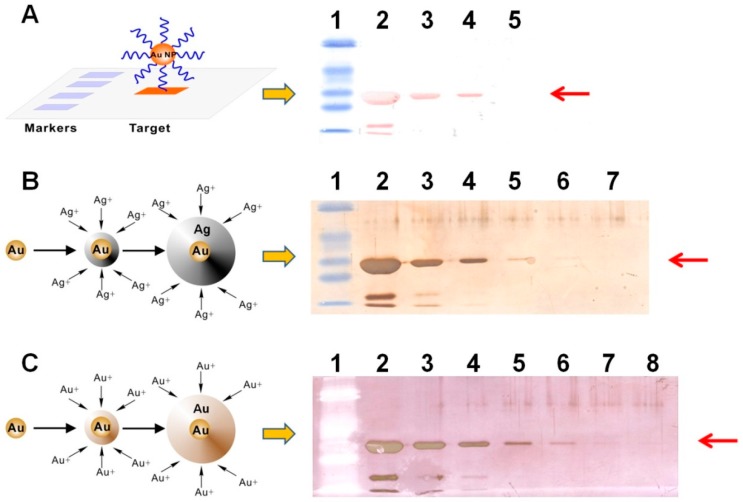
Western blot analysis of human α-thrombin in PBS buffer using Apt-AuNP labeling (**A**), Apt-AuNP labeling followed by silver enhancement (**B**), and Apt-AuNP labeling followed by gold enhancement (**C**). Lane 1 contained pre-stained low-range protein standards. Lanes 2–8 contained varying amounts of human α-thrombin: 5 µg (lane 2), 1 µg (lane 3), 500 ng (lane 4), 100 ng (lane 5), 50 ng (lane 6), 10 ng (lane 7), and 5 ng (lane 8). The red arrows indicate α-thrombin.

To select a suitable signal amplification strategy for naked-eye protein detection on membrane, we first compared the light-scattering signals generated from Apt-AuNPs, Apt-AuNPs with silver enhancement, and Apt-AuNPs with gold enhancement (Figure 2). Figure 2A shows a typical image of membrane blotted human α-thrombin (diluted in PBS buffer) after labeling with Apt-AuNPs. The light-scattering intensities from Apt-AuNPs labeled thrombin are readily distinguishable from the background, resulting in intense red protein bands (36 kD) on the membrane. The products of the band sizes and band intensities are proportional to the amount of thrombin in the range of 500 ng to 5 µg. The light-scattering signals from Apt-AuNP labels were further amplified by depositing silver or gold onto them through a catalytic reduction of silver (Figure 2B) or gold ions (Figure 2C). By amplifying the signal with silver enhancement, we were able to visually identify as low as 50 ng thrombin on the membrane (Figure 2B). Gold enhancement further improved detection sensitivity by forming larger microstructures [9,10], resulting in a detection limit of 5 ng thrombin (Figure 2C). Thus, we chose gold enhancement to amplify the detection signal for Apt-AuNPs in the following studies.

**Figure 3:**
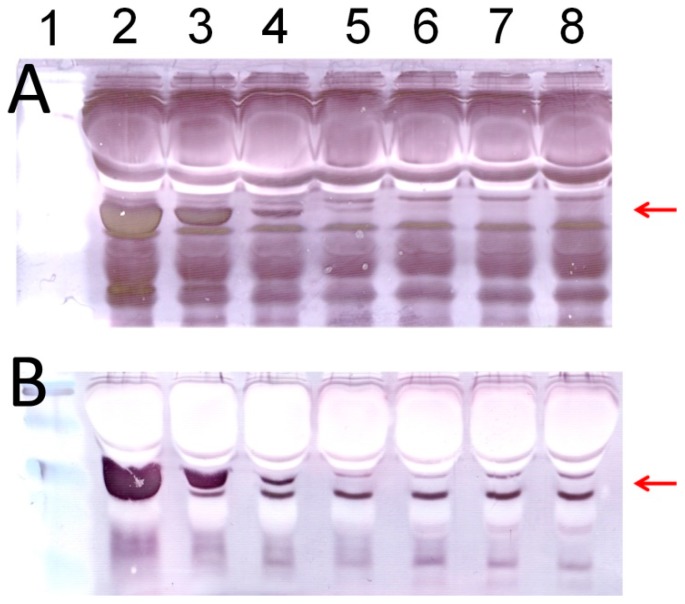
Western blot analysis of human α-thrombin in a 10-time diluted human serum sample: (**A**) protein detection using unprotected Apt-AuNP labeling followed by gold enhancement; (**B**) protein detection using a PolyA-oligo10 protected Apt-AuNP probe followed by gold enhancement. Lane 1 contained pre-stained low-range protein standards. Lanes 2–8 contained varying amounts of human α-thrombin: 5 µg (lane 2), 1 µg (lane 3), 500 ng (lane 4), 100 ng (lane 5), 50 ng (lane 6), 10 ng (lane 7), and 5 ng (lane 8). The red arrows indicate α-thrombin.

**Figure 4:**
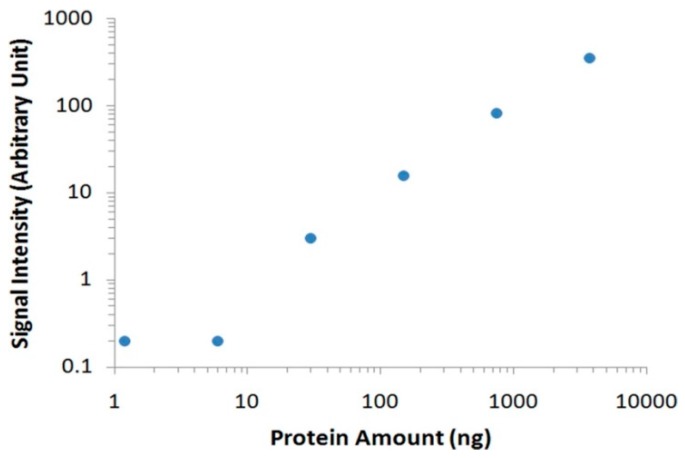
Quantitative protein analyses using targeted enlargement of Apt-AuNPs. To achieve the quantitative information, the PVDF membrane that has been blotted with proteins and undergone the targeted-enlargement was scanned into a computer using a desktop flatbed scanner. The resulting colored digital image was converted into a grayscale image using Adobe Photoshop. The signal intensity of each protein band was then calculated as the product of the band area (mm^2^) and the darkness (0–256).

Having established the signal amplification method for Apt-AuNP labels, we further applied this method to analyze thrombin spiked in human serum. After separating with gel electrophoresis and transferring onto PVDF membranes, target thrombin and interfering serum proteins were incubated with Apt-AuNPs followed by a subsequent gold enhancement. Figure 3 shows typical images of protein analyses using Apt-AuNP labeling and gold enhancement on PVDF membranes. In the absence of the protection DNA (Figure 3A), Apt-AuNP labeling and subsequent gold enhancement was able to amplify the detection signal for human α-thrombin in the diluted human serum, indicated by the protein bands at molecular weight of 36 kD. However, there is a strong background resulting from interfering serum proteins. This observation is consistent with the previous report that high concentrations of matrix proteins, e.g., 8% human plasma, could interfere with the metal enhancement of Apt-AuNPs for protein detection [12]. However, by using the competitively protected Apt-AuNPs, we could effectively eliminate nonspecific protein bindings, resulting in an amplified scattering signal specifically for the target thrombin (Figure 3B). By eliminating the nonspecific bindings from interference serum proteins, we could easily identify the target thrombin without the need of any instrument. To further analyze the data with imaging software, we scanned the membrane with a commonly used desktop flatbed scanner. Quantitative information could then be obtained by calculating the areas and intensities of protein bands using Adobe Photoshop software (Figure 4), and as low as 30 ng of thrombin was detected in human serum samples.

## 4. Conclusions 

We have successfully developed a target enlargement strategy of Apt-AuNPs for colorimetric detection of a minute amount of proteins in human serum samples. The success of developing this amplification strategy demonstrates (1) the extremely high selectivity of our competitively protected Apt-AuNP probes to target proteins over a complicated sample matrix and interfering proteins; (2) the compatibility of our competitively protected Apt-AuNP probes to other signal amplification strategies. These features make our strategy ideal to be applied in diverse sensor formats to achieve targeted signal amplification for protein analyses.

## Abbreviations

The following abbreviations are used in this manuscript:

Apt-AuNP: Aptamer-functionalized gold nanoparticle
SRM: Selected Reaction Monitoring
MRM: Multiple Reaction Monitoring
SDS-PAGE: Sodium dodecyl sulfate polyacrylamide gel electrophoresis
BSA: Bovine serum albumin
TCEP: Tris (2-carboxyethyl) phosphine hydrochloride
